# Bacterial Puppeteering: How the Stealth Bacterium *Coxiella* Pulls the Cellular Strings

**DOI:** 10.3390/pathogens14090896

**Published:** 2025-09-05

**Authors:** Dylan Ruart, Juliette Riedinger, Sihem Zitouni, Arthur Bienvenu, Matteo Bonazzi, Eric Martinez

**Affiliations:** Institut de Recherche en Infectiologie de Montpellier (IRIM), Université de Montpellier, CNRS, CEDEX 5, 34293 Montpellier, France; dylan.ruart@irim.cnrs.fr (D.R.); juliette.riedinger@irim.cnrs.fr (J.R.); zsihem.szitouni@gmail.com (S.Z.); art.bienvenu@gmail.com (A.B.); matteo.bonazzi@irim.cnrs.fr (M.B.)

**Keywords:** *Coxiella burnetii*, host–pathogen interactions, effector proteins

## Abstract

*Coxiella burnetii*, the causative agent of Q fever, is a highly infectious pathogen capable of invading diverse cell types, from alveolar macrophages to trophoblasts. Within host cells, it establishes a replicative niche named *Coxiella*-containing vacuole (CCV). This is driven by effector proteins secreted by the bacterium into the host cell cytoplasm via a Type 4b Secretion System (T4SS). Advances in axenic culture and mutagenesis allowed the characterization of *Coxiella* effector proteins, revealing their host targets and strategies of cellular subversion. This review highlights recent insights into *Coxiella* effector proteins and their manipulation of host processes, from vesicular trafficking to innate immunity.

## 1. Introduction

In the intricate interplay between pathogens and their hosts, some bacterial species have evolved a remarkable ability to manipulate host cells, bending cellular processes to serve their own survival and replication. Much like a skilled puppeteer pulling strings, these bacteria employ a sophisticated arsenal of molecular tools to take control of the host’s cellular machinery. One such master of microbial puppeteering is *Coxiella burnetii*, the etiological agent of Q fever, a zoonotic disease with variable clinical manifestations ranging from acute febrile illness to life-threatening chronic infections like endocarditis [1].

*Coxiella* takes over infected cells by establishing a unique lysosome-derived compartment, the *Coxiella*-containing vacuole (CCV), in which it replicates [2]. Unlike many intracellular pathogens that escape the lysosomal pathway, *Coxiella* not only tolerates but thrives in the highly acidic and degradative environment of the CCV. To orchestrate the biogenesis and maintenance of this replicative niche, *Coxiella* employs a type IV secretion system (T4SS). This molecular apparatus delivers a repertoire of effector proteins directly into the host cytoplasm to reprogram cellular functions [2] (Table 1 and Table 2).

The effector proteins secreted by *Coxiella* are the instruments of control, targeting a wide range of host cell processes. By modulating membrane trafficking, autophagy, apoptosis, and immune signaling, these effectors ensure the bacterium’s survival, replication, and evasion of host defenses. Many of these proteins mimic host regulatory elements, manipulating cellular pathways in a manner so precise that it recalls the dexterity of a master puppeteer. For example, certain effectors manipulate RAB GTPases to control vesicle trafficking, while others interact with autophagic machinery to maintain the CCV. This strategic hijacking underscores the sophistication of *Coxiella*’s intracellular lifestyle and the pivotal role of effector proteins in its pathogenicity.

The discovery of *Coxiella* effectors has been a journey shaped by technological innovation [2]. Initial genomic sequencing provided the first glimpse of the effector repertoire, identifying numerous genes encoding proteins with secretion signals [41,42]. Early functional studies, often conducted using surrogate systems like *Legionella pneumophila*, demonstrated that *Coxiella* effectors could functionally mimic those of other intracellular pathogens [43]. Subsequent proteomic and transcriptomic analyses of infected cells revealed additional effector candidates, many of which have since been experimentally validated [44,45]. These studies have illuminated the multifaceted strategies by which *Coxiella* reconfigures host cellular processes to its advantage.

Despite this progress, much remains to be uncovered about the full extent of *Coxiella*’s capacity to control the host cell. Recent advances in high-throughput screening, structural biology, and host–pathogen interaction studies have expanded our understanding of effector biology, revealing new mechanisms by which these proteins influence host cell fate. By continuing to unravel the strings of this microbial puppeteer, we gain not only insights into the biology of *Coxiella* but also broader knowledge about host–pathogen interactions and the vulnerabilities of cellular systems that bacteria exploit.

This review explores the mechanisms of *Coxiella*’s effector proteins, focusing on their roles in subverting host processes and maintaining the intracellular niche. By examining how these proteins manipulate host cells, we aim to illuminate the complex molecular dialogue that defines *Coxiella* pathogenesis. Additionally, we discuss recent advances and emerging questions in the study of bacterial effectors, providing a comprehensive overview of *Coxiella*’s sophisticated control of its host.

## 2. Discovery and Characterization of *Coxiella burnetii* Effector Proteins

The identification of *Coxiella burnetii* effector proteins has been a gradual process, closely linked to technological advances in microbiology, molecular genetics, and bioinformatics. Early research into *Coxiella* pathogenesis suggested the involvement of a specialized secretion system to manipulate host cell processes, but progress was initially hindered by the bacterium’s obligate intracellular nature and the lack of genetic tools for its study.

### 2.1. Early Indications and Genomic Insights

The first significant breakthroughs came from genomic sequencing of *Coxiella*. The complete genome sequence of the *Coxiella* RSA 493 strain, published in 2003, revealed the presence of genes encoding a type IV secretion system (T4SS) similar to the Dot/Icm system in *Legionella pneumophila* [41]. This discovery suggested that *Coxiella* utilized a comparable mechanism to translocate effector proteins into host cells. Comparative genomics highlighted the conservation of T4SS components, sparking investigations into whether *Coxiella* also deployed a large effector repertoire to manipulate host processes [46].

### 2.2. Effector Identification via Bioinformatics and Surrogate Systems

With the T4SS system established as a central virulence factor, researchers turned to bioinformatics to predict potential effector proteins. Studies utilized algorithms to identify proteins with eukaryotic-like motifs, T4SS secretion signals, and other features indicative of host cell targeting [42,47]. One of the earliest approaches involved screening *Coxiella* genes for their ability to rescue phenotypes in *L. pneumophila* mutants lacking specific effectors, a surrogate system for studying Dot/Icm-dependent secretion [43,46]. This provided strong evidence that *Coxiella* effectors shared functional similarities with those of other intracellular pathogens. The development of high-throughput omics technologies enabled researchers to systematically study *Coxiella* effector candidates. Proteomic analyses of infected host cells and purified *Coxiella* compartments identified proteins enriched during intracellular growth [48,49]. Similarly, transcriptomic studies identified genes upregulated during intracellular replication [50,51].

### 2.3. High-Throughput Screening and Genetic Manipulation

The advent of high-throughput translocation assays represented a significant leap forward in effector identification. One widely used method, the beta-lactamase translocation assay, tested the ability of *Coxiella* proteins to translocate into host cells when fused to a reporter enzyme. This technique enabled the identification of dozens of effectors in a single experiment, rapidly expanding the known effector repertoire [52]. In parallel, advances in genetic tools for *Coxiella*, such as transposon mutagenesis [3,20,53] and targeted gene knockouts [54,55], allowed researchers to study the functions of individual effectors in host cell manipulation. In a recent study, a CRISPR-Cas9 method based on cytosine base editing was applied to facilitate targeted genetic modifications in *Coxiella* [56]. In parallel, CRISPR interference (CRISPRi) was also adapted to *Coxiella* to repress gene expression [11,56,57]. These tools enabled the generation of double mutants, such as those deficient in both *cig57* and *cig2*, revealing their independent roles in vacuole formation and bacterial replication. This advancement significantly enhances the genetic toolkit for studying *Coxiella* pathogenesis.

## 3. Manipulation of Host Cell Endosomal Trafficking

**Figure 1:**
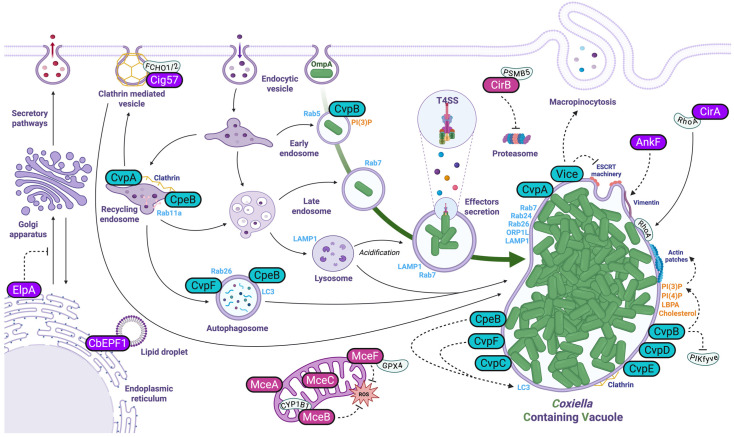
Manipulation of endosomal trafficking.

The biogenesis of *Coxiella*-containing vacuoles is essential for its intracellular replication. In order to establish this replicative niche, the bacterium secretes effector proteins to manipulate vesicular trafficking pathways (Figure 1). The seminal articles by Larson and colleagues [4,21] represent a milestone in understanding *Coxiella burnetii* pathogenesis, as they were the first to identify *Coxiella* vacuolar proteins (Cvps) as critical effectors in the biogenesis and maintenance of the *Coxiella*-containing vacuole (CCV). Using bioinformatics approaches, the study screened for Dot/Icm type IV secretion system substrates with features such as eukaryotic-like motifs, identifying several candidates, including CvpA, CvpB, and CvpC [4,21]. Localization studies revealed that these effectors are translocated into host cells and localize to the CCV, endosomes, and other vesicular compartments. Over the past 10 years, important advances in the characterization of Cvps and the identification of other effector proteins demonstrated how the manipulation of membrane traffic in infected cells is key for the development of CCVs, with important consequences in virulence [2].

Schematic illustration of the *Coxiella* internalization and maturation of the *Coxiella*-containing vacuole (CCV) resulting from manipulation of host cell trafficking pathways. *Coxiella* enters host cells via phagocytosis or receptor-mediated endocytosis, facilitated by the invasin OmpA. The nascent *Coxiella*-containing vacuole (CCV) progresses through the degradative endocytic pathway, sequentially fusing with early endosomes (EE) and late endosomes (LE). Upon exposure to the acidic, degradative environment, *Coxiella* transitions to its replicative Large Cell Variant (LCV) form and activates the Dot/Icm type IV secretion system (T4SS). This system translocates > 130 effector proteins into the host cytosol. These effectors hijack host processes through three primary mechanisms: (1) Vacuole remodeling (purple), where select effectors transform the CCV into a highly fusogenic compartment that supports bacterial proliferation,(2) metabolic manipulation (pink), through which a subset targets organelles (e.g., mitochondria) to alter host metabolism and suppress defense responses, and (3) membrane fusion (blue), whereby specific effectors mediate CCV fusion with clathrin-coated vesicles, autophagosomes, and endolysosomal vesicles to establish the expanded replicative niche.

### 3.1. Effector Proteins Targeting Membrane Trafficking and Endosomal Sorting

Several effector proteins secreted by the Dot/Icm type IV secretion system (T4SS) target specific steps in endosomal sorting and membrane trafficking to establish and sustain the CCV (Figure 1). Among these, *Coxiella* vacuolar proteins (Cvps) are effector proteins that localize at CCV membranes and are essential to their biogenesis [4,19,21]. CvpA plays a pivotal role in the biogenesis of CCVs, which is crucial for the bacterium’s replication within host cells. CvpA is characterized by multiple endocytic sorting motifs, including dileucine and tyrosine-based motifs, which facilitate its interaction with clathrin adaptor protein complexes such as AP1, AP2, and AP3 [21]. These interactions are essential for the localization of CvpA to the CCV membrane and endocytic recycling vesicles. The deletion of the *cvpA* gene in *Coxiella* results in impaired replication and CCV development, underscoring its importance in infection. Ectopic expression of CvpA in host cells reveals its localization to tubular and vesicular domains associated with endocytic recycling, and mutations in its sorting motifs disrupt these interactions [21]. Furthermore, depletion of AP2 or clathrin significantly inhibits *Coxiella* replication, highlighting the critical role of clathrin-mediated vesicle trafficking in CCV formation and maintenance [21].

CvpC, also known as Cig50, localizes to the parasitophorous vacuole (PV) membrane and to LAMP1-positive vesicles, indicating its involvement in the modulation of host cell endocytic and vesicular trafficking pathways [4]. Studies have shown that CvpC, along with other effectors such as CvpB, CvpD, and CvpE, plays a crucial role in promoting the intracellular replication of *Coxiella* [4]. When ectopically expressed as fluorescently tagged fusion proteins, CvpC labels the PV membrane, suggesting its direct interaction with this compartment. Mutants of *Coxiella* lacking the *cvpC* gene exhibit significant defects in intracellular replication and PV formation, highlighting the importance of CvpC in the pathogen’s virulence strategy. Notably, the growth of *Coxiella cvpC* mutants can be rescued when these mutants cohabitate with wild-type bacteria in a common PV, indicating that CvpC contributes to the collective effort of multiple effectors in creating a replication-permissive environment within the host cell [4]. Additionally, CvpC has been observed to exhibit partial localization with the transferrin receptor, a marker of recycling endosomes. However, this association does not perturb the uptake of transferrin, suggesting that CvpC’s role is more nuanced and does not disrupt the basic functions of recycling endosomes but rather modulates them to facilitate *Coxiella*’s intracellular survival and replication [4]. Overall, CvpC is a key effector protein that targets the PV membrane and regulates essential host cell processes to promote the successful intracellular replication of *Coxiella*. Interestingly, it has been shown that the expression of CvpD is regulated by the small noncoding RNA CbsR12, which binds to and downregulates the translation of *cvpD* transcripts in mammalian cell cultures [58]. This hints at a possible additional system for the regulation of *Coxiella* effector proteins.

Similar to Cvps, Cig57 localizes at the CCVs and other structures in the cytoplasm. It specifically interacts with FCHO1/2 proteins, key components in clathrin-mediated endocytosis, effectively hijacking this vesicular trafficking pathway. This interaction enables *Coxiella* to modulate endocytic processes, promoting the biogenesis of the *Coxiella*-containing vacuole (CCV), which serves as its replicative niche. Mutant strains of *Coxiella* lacking Cig57 exhibit significant defects in intracellular growth, underscoring the effector’s vital role in the pathogen’s lifecycle [34,35].

Finally, Graham and colleagues suggested that ER-localizing protein A (ElpA, CBUD1884) might be a Dot/Icm substrate found in certain *C. burnetii* isolates that could localize to the ER and disrupt the host cell secretory transport [59]. Testing its Dot/Icm dependency in *Coxiella* strains and the analysis of *elpA* knockout mutants would shed light on the role of ElpA in *Coxiella* virulence.

### 3.2. Effector Proteins Manipulating Lipid Metabolism

Intravacuolar bacterial pathogens have adapted to an intracellular lifestyle by triggering the biogenesis of replicative niches, whose protein and lipid composition is dictated by the subversion of host membrane trafficking by bacterial effector proteins. In this context, host membrane lipids and phosphoinositides (PIs) have emerged as new targets for several intracellular bacterial pathogens. PIs are short-lived lipids whose spatiotemporal localization is determined by the activity of specific kinases and phosphatases. Together with RAB GTPases, their localization dictates the identity of cellular membranes and organelles and allows the targeted recruitment and activation of many downstream proteins, making them important signaling hubs for the regulation of cellular functions, including membrane traffic, actin rearrangement, and immunity [60]. Bacterial effectors use host PIs as anchors to target specific host cell membranes and manipulate their metabolism directly, by mimicking eukaryotic kinases or phosphatases, or indirectly, by enhancing or blocking the activity of host PI-metabolizing enzymes [61]. Overall, the manipulation of lipids and PI metabolism by bacterial proteins has pleiotropic effects on downstream signaling pathways, which are collectively beneficial for the establishment/development of infections. A key factor in CCV development is the regulation of cholesterol within the CCV membrane, as excessive cholesterol accumulation can lead to increased acidification and bacterial death [62]. To modulate cholesterol levels within the CCV, *Coxiella* expresses a sterol-modifying protein known as Stmp1 [63,64]. Stmp1 is homologous to eukaryotic sterol reductases and is believed to have been acquired through horizontal gene transfer from amoebal hosts. While *Coxiella* lacks the complete enzymatic pathway for de novo cholesterol biosynthesis, Stmp1 appears to play a pivotal role in modifying host-derived sterols to maintain a conducive environment for bacterial replication [63,64]. Interestingly, Stmp1 is not an effector protein, but it localizes at the bacterial cell membrane.

CvpB has been characterized as the first *Coxiella* effector capable of directly interacting with lipids (lipid-interacting effectors, or LIEs). These bacterial effector proteins are capable of interacting with host cell lipids in order to modify their localization, abundance, or nature [65]. CvpB plays a crucial role in the biogenesis and maintenance of the CCV [3,5]. This effector interacts with phosphoinositides, specifically binding to phosphatidylinositol 3-phosphate [PI(3)P] and phosphatidylserine (PS) on CCVs and early endosomal compartments [5]. By modulating the activity of phosphatidylinositol 5-kinase PIKfyve, CvpB alters PI(3)P metabolism, leading to an accumulation of PI(3)P at the CCVs. This interaction is vital for the optimal fusion of autophagosome-derived membranes with CCVs, promoting an adequate environment for bacterial survival and replication [5,66]. Mutations in the *cvpB* gene result in a multivacuolar phenotype, indicating defects in CCV biogenesis, which can be rescued by gene complementation [3,5]. Interestingly, intracellular replication is not affected by mutations in *cvpB*, however, studies in model organisms like SCID mice and *Galleria mellonella* have demonstrated that *cvpB* mutations lead to attenuated virulence, underscoring the importance of CCV biogenesis (regardless of bacterial replication) in pathogenesis [6,66]. In 2025, Bird and colleagues highlighted the role of CvpB in modulating the host lysosomal environment to support bacterial replication [7]. CvpB appears to be involved in the selective removal of cathepsin B, a lysosomal protease whose proteolytic activity poses a threat to the bacterium within the CCV. Although CvpB alone is not sufficient to drive cathepsin B removal when ectopically expressed, its absence results in altered vacuole morphology, suggesting that CvpB helps to develop an optimal vacuolar environment by stimulating protease exclusion. Notably, this effect appears to be independent of CvpB’s known role in phosphoinositide modulation via PIKfyve inhibition [5].

Similar to CvpB, CvpE also binds PI(3)P and outcompetes PIKfyve for lipid binding, thus inhibiting the activity of the lipid kinase. However, differently from CvpB, the ectopic expression of CvpE in cells leads to the formation of large Lysosome-Like Vacuoles (LLVs) positive for autophagosomal markers [37]. In the context of infection, *Coxiella* Δ*cvpE* mutants exhibit significant defects in intracellular replication and PV formation, with genetic complementation rescuing growth and PV generation. CvpE could maintain the CCV architecture, preventing fission and promoting intracellular bacterial replication [4]. This phenotype could be mediated by the CvpE-dependent inhibition of lysosomal calcium channel TRPML1 activity, leading to lysosomal fission defects. Accordingly, TRPML1 agonists restore normal vacuole size in cells expressing CvpE. Interestingly, the TRPML1 agonist ML-SA5 restricts CCV development and bacterial replication *in cellulo*, hinting at a possible host-targeting antimicrobial therapy [37]. The fact that CvpE was found enriched on mitochondria isolated from *Coxiella*-infected THP1 cells [25] indicates that CvpE might act on different organelles to ensure optimal CCV biogenesis.

Following the identification of the first *Coxiella* Lipid-Interacting Effectors (LIEs), a screen using lipid-binding probes revealed that, other than PI(3)P, phosphadidylinositol-4-phosphate (PI(4)P) and lysobisphosphatidic acid (LBPA) are also actively recruited at CCVs by effector proteins, whereas PI(3,5)P_2_ and phosphatidylserine (PS) are integral components of CCVs, likely resulting from the maturation of CCVs along the endocytic maturation pathway [38]. A phenotypic screen of a library of *Coxiella* transposon mutants identified the effector Vice (for Vacuole Inducing *Coxiella* Effector, encoded by the gene *cbu2007*) as responsible for the recruitment of LBPA at CCVs [38]. Vice interacts with specific host cell lipids, including LBPA and PS, and is required for the initial expansion of CCVs. Ectopic expression of Vice in cells leads to the formation of VICs (Vice-induced compartments), large intracellular vacuoles highly resembling CCVs in infected cells. VIC biogenesis is driven by a double activity of Vice, which stimulates macropinocytosis and perturbs the Endosomal Sorting Complex Required for Transport (ESCRT) machinery, stabilizing these compartments by potentially disrupting the formation of intraluminal vesicles (ILVs) [38].

The recently identified *Coxiella* effector protein CBU1370, renamed CbEPF1, is another key factor in host lipid metabolism manipulation. CbEPF1 contains two FFAT motifs, facilitating its interaction with VAP proteins in the endoplasmic reticulum (ER). Initially localizing to the ER, CbEPF1 translocates to lipid droplets (LDs) during their biogenesis, promoting their growth by establishing ER–LD contact sites [30]. This process is dependent on the integrity of the FFAT motifs, highlighting their role in CbEPF1’s function. These findings reveal a novel mechanism by which LDs may serve as critical metabolic reservoirs or signaling platforms that facilitate bacterial replication within host cells.

### 3.3. Effector Proteins Influencing Autophagy and Host Vesicle Fusion

Autophagy, a conserved cellular process for degrading and recycling cellular components, plays a dual role in infections: it can act as a host defense mechanism or be exploited by intracellular pathogens. In the case of *Coxiella*, this process is co-opted to promote bacterial survival and replication within host cells. *Coxiella* uniquely thrives in a lysosome-like compartment, which is acidic and nutrient-rich. It has been shown that *Coxiella* utilizes the host’s autophagic machinery to remodel this vacuole and maintain its biogenesis [67]. Early studies demonstrated that markers of autophagy, such as microtubule-associated protein 1A/1B-light chain 3 (LC3), are recruited to the CCV. This recruitment suggests that autophagosomes or autophagic components contribute to the expansion of the CCV [68,69]. The interaction with autophagy is not merely coincidental. Pharmacological or genetic inhibition of autophagy, such as through the depletion of autophagy-related proteins (e.g., Atg5, Atg7), results in decreased CCV size and impaired bacterial replication [70,71]. This finding underscores the importance of autophagy in *Coxiella*’s intracellular lifecycle. Conversely, treatments that enhance autophagy, such as starvation or certain drugs, promote CCV expansion and increased bacterial replication. The ability of *Coxiella* to harness autophagy represents a unique adaptation among intracellular pathogens. While many bacteria evade or inhibit autophagy as a host defense, *Coxiella* relies on it to establish its niche. This reliance reflects the dual nature of autophagy: while it can serve as a mechanism to restrict bacterial growth, *Coxiella* has evolved to convert it into a survival and replication advantage.

Among effector proteins manipulating autophagy, the plasmid-encoded effector protein CpeB plays a pivotal role in the pathogenesis of *Coxiella* by manipulating host cell autophagy to facilitate bacterial replication. CpeB is secreted via the Dot/Icm type IV secretion system (T4SS) and localizes to autophagosomes, suggesting its involvement in modulating autophagic pathways [39]. It promotes the accumulation of LC3-II, a marker of autophagy, through a pathway involving the host GTPase RAB11a, which is crucial for CCV development and bacterial proliferation. Studies have shown that *Coxiella* strains lacking the QpH1 plasmid, which encodes CpeB, exhibit reduced LC3-II accumulation, smaller CCVs, and lower bacterial loads in THP-1 cells. However, expressing CpeB in these strains restores LC3-II levels but does not affect CCV size. In an SCID mouse model, infections with strains expressing CpeB result in significantly higher bacterial burdens in the spleen and liver compared to strains lacking this effector [72]. This highlights CpeB’s role in enhancing virulence and underscores its importance in modulating autophagy to promote *Coxiella* survival and replication within host cells. Collectively, these findings suggest that CpeB is critical for the successful manipulation of host cellular processes necessary for the pathogen’s lifecycle and pathogenicity.

Among Cvps, the effector protein CvpF plays a crucial role in the biogenesis of the CCV and the bacterium’s virulence. CvpF localizes to vesicles with autolysosomal features and CCVs, where it interacts with the host small GTPase RAB26. This interaction leads to the recruitment of the autophagosomal marker LC3 to CCVs, a process essential for optimal vacuole biogenesis and bacterial replication [19]. Mutants lacking the *cvpF* gene (*cvpF::Tn*) exhibit severe replication defects in epithelial cells and reduced virulence in the SCID mouse model, highlighting the importance of CvpF in *Coxiella*’s intracellular survival and pathogenicity. Complementation of the *cvpF::Tn* mutation restores the expression of CvpF and partially rescues the replication defect both in vitro and in vivo [19]. CvpF colocalized with the lysosomal marker LAMP1 at CCVs, and its secretion was validated using a β-lactamase secretion assay, demonstrating that CvpF is indeed a Dot/Icm secreted protein. Overall, CvpF manipulates endosomal trafficking and macroautophagy to facilitate the development of the CCV, underscoring its critical role in *Coxiella*’s intracellular niche formation and virulence.

Finally, in addition to its role in phosphoinositide metabolism, CvpB influences autophagosome-derived membrane fusion with the CCV, promoting vacuole expansion [5]. The recruitment of autophagic machinery by CvpB demonstrates the multifaceted strategies employed by *Coxiella* to co-opt autophagy for intracellular survival.

### 3.4. Eukaryotic-like Effectors

One striking feature of *Coxiella* is its extensive arsenal of eukaryotic-like genes (EUGENs), which encode proteins with domains that structurally or functionally resemble eukaryotic counterparts [73]. EUGENs were likely acquired via horizontal gene transfer, possibly from amoebal hosts, supporting the idea that environmental interactions shaped the bacterium’s current pathogenic lifestyle. Bioinformatics analysis identified a broad repertoire of EUGENs in *Coxiella*, many of which encode type IV secretion system (T4SS) effectors [27]. These include proteins with ankyrin repeats, F-box domains, and coiled-coil motifs—hallmarks of host-interacting modules. These effectors are thought to be instrumental in host cell manipulation, allowing *Coxiella* to mimic or interfere with eukaryotic processes to facilitate intracellular survival. Several EUGENs localize to distinct host organelles and compartments, hinting at specialized roles in modulating trafficking, signaling, and cytoskeletal dynamics [27]. This molecular mimicry strategy underscores the sophistication of *Coxiella*’s host adaptation and pathogenesis.

Among EUGENs the effector protein AnkF is important for *Coxiella* intracellular replication. Indeed, an *ankF* knockout mutant retains the capacity of invading host cells comparably to wild-type bacteria but exhibits impaired intracellular replication, suggesting AnkF’s involvement in establishing the replicative CCV [15]. Through yeast two-hybrid screening, vimentin was identified as an AnkF interactor. Ectopically expressed AnkF partially localized around the CCV, and endogenous vimentin was recruited to the CCV in a time-dependent manner. However, siRNA-mediated knockdown of vimentin did not affect bacterial replication, indicating potential redundancy with other cytoskeletal components [15]. These findings highlight AnkF’s essential role in CCV development, though its precise mechanism remains to be elucidated.

### 3.5. Coxiella Effectors and the Manipulation of the Host Cell Cytoskeleton

The host cell cytoskeleton, particularly actin filaments and microtubules, plays a crucial role throughout the intracellular life cycle of *Coxiella burnetiid* [74,75]. During the early stages of infection, actin remodeling facilitates bacterial internalization via phagocytosis or micropinocytosis [76]. Once inside the host cell, the nascent CCV traffics along microtubules toward the perinuclear region, where it matures into a spacious, fusogenic compartment conducive to bacterial replication. Disruption of microtubules, for example with nocodazole, impairs CCV positioning and expansion, highlighting the dependence of CCV biogenesis on intact microtubule tracks [75]. Actin filaments also contribute to CCV stability and dynamics, cortical actin helps maintain the structural integrity of the vacuole and mediates interactions with host organelles. CirA is a key *Coxiella burnetii* effector protein, characterized by several “arginine finger-like motifs”, which activate the small GTPase RhoA [76]. By stimulating RhoA, CirA disrupts the host cell’s cytoskeleton by breaking stress fibers, thereby altering cell structure. This activity promotes the formation and enlargement of the vacuole containing *Coxiella* bacteria (CCV), which facilitates bacterial replication [8]. In addition, CirA is involved in vesicular trafficking within the host cell, as suggested by its endosome-lysosome-basolateral sorting signals. Unlike other bacteria that use GTPases to facilitate their entry into host cells, CirA promotes the development of the bacterial vacuole once the bacteria are already inside [8]. CirA directly stimulates RhoA, promoting its GTPase activity, which leads to a reorganization of the host cytoskeleton to promote biogenesis and enlargement of the vacuole containing *Coxiella*. In addition, several other pathogenic bacteria, such as *Yersinia pseudotuberculosis* [77], *Salmonella enterica* [78], *Legionella pneumophila* [79], and *Vibrio cholerae* [80], have effectors that also target Rho GTPases to modulate the host cytoskeleton and facilitate their own survival and infection.

## 4. Manipulation of Cell Metabolism

Mitochondria play a central role in cellular metabolism, innate immunity, and programmed cell death such as apoptosis and pyroptosis, making them a strategic target for intracellular pathogens such as *Coxiella burnetii*. Recent studies have identified a growing cohort of *Coxiella* effector proteins that localize to mitochondria during infection, suggesting that subversion of mitochondrial function is a key aspect of the pathogen’s intracellular survival strategy.

To date, at least six *Coxiella* effectors, MceA through MceF, have been reported to associate with mitochondrial compartments at various stages of infection. Among these, MceA (CBU0077) is farnesylated on a C-terminal cysteine residue and forms multimeric complexes on the mitochondrial outer membrane [10]. However, the role of MceA in *Coxiella* pathogenesis remains to be defined, as *mceA*::Tn mutants are not affected in their capacity to form CCVs and replicate either in epithelial HeLa cells or THP1 myeloid cells.

Subcellular fractionation combined with mass spectrometry has further revealed mitochondrial localization for MceB (CBU0937), MceC (CBU1425), MceD (CBU1594), and MceE (CBU1677) during *Coxiella* infection [25]. Among these, MceB has garnered particular attention due to its interaction with the mitochondrial cytochrome P450 enzyme CYP1B1, an interaction that may influence mitochondrial lipid metabolism or redox signaling [26]. However, the study by Yang and colleagues suggested that MceB is a *Coxiella* outer membrane protein presenting predicted structure homology to porins and might participate in nutrient acquisition from the CCV lumen [81]. A hypothetical model could reconcile the two localizations observed for MceB: once in *Coxiella* periplasm, this effector could either be inserted in the outer membrane of the bacteria or interact with the T4BSS inner-membrane-associated protein DotF, allowing its secretion [11].

MceC demonstrates a more defined spatial distribution, localizing specifically to the mitochondrial inner membrane. There, it is associated with components of the organelle’s proteostasis machinery, including the ATP-dependent protease YME1L, which is integral to the regulation of mitochondrial protein quality control and dynamics [25]. This suggests a potential role for MceC in modulating mitochondrial stress responses or protein turnover.

MceF (CBU1543) represents another effector that actively recruits host cell machinery to the mitochondria. Specifically, MceF has been shown to mobilize Glutathione Peroxidase 4 (GPX4), a key antioxidant enzyme, to the organelle [82]. This interaction may serve to mitigate host-derived oxidative stress during infection, thereby creating a more permissive environment for bacterial replication.

Collectively, these findings underscore the multifaceted strategy employed by *Coxiella burnetii* to manipulate mitochondrial biology. While the precise molecular mechanisms and functional implications of these effector–mitochondria interactions remain to be fully elucidated, they offer valuable insights into the pathogen’s capacity to modulate host cell fate, immune signaling, and metabolic state. Future studies will be essential to define the biochemical activities of these effectors and to determine how they collectively influence mitochondrial function and contribute to *Coxiella*’s intracellular lifecycle.

Recently, the *Coxiella* effector protein CirB (encoded by CBU0425) has been shown to play a crucial role in modulating host cell functions during infection. By using affinity tag purification mass spectrometry (AP-MS) to construct a comprehensive protein–protein interaction (PPI) map, Fu and colleagues identified CirB’s interaction with the 20S core proteasome in human cells. Specifically, CirB binds to the PSMB5 subunit, inhibiting the proteasome’s hydrolytic activity. This inhibition impairs the host cell’s ability to degrade proteins, which is essential for the replication of *Coxiella* within the host cell [14].

## 5. Subversion of Host Innate Immunity/Signaling

During the early phases of infection, pathogens activate innate immune cells such as macrophages, cytotoxic natural killer (NK) cells, and antigen-presenting dendritic cells (DCs), which initiate specific immune responses through antigen processing and presentation to cells of the adaptive immune response, such as T and B lymphocytes [83]. Microbial detection is crucial to ensure the elimination of pathogens. Pattern recognition receptors (PRRs) sense a broad range of pathogen-specific molecules termed pathogen-associated molecular patterns (PAMPs) at the host cell surface as well as in the cytosol [84] and sense damage-associated molecular patterns (DAMPs) released by damaged host cells and tissues [85]. Stimulated receptors activate a series of signaling pathways leading to increased production of interferon I (IFN-I), proinflammatory cytokines. A common strategy of persistent bacterial pathogens is to evade immune clearance by overcoming cellular functions. Some bacterial effector proteins are secreted to promote persistence by modulating host innate immune responses and interfering with host detection mechanisms, signaling pathways, or host transcription and translation. While some pathogens exploit inflammation to their own advantages, others, such as *Coxiella*, prevent the recognition by the innate immune system to promote their survival [86]. Studies on the manipulation of host innate immunity by *Coxiella* appear controversial, as they report diverging results according to bacterial strains and host cells used. The ability of *Coxiella* to alter the immune response has nevertheless been demonstrated in many ways, showing the importance of investigating how the pathogen subverts host defenses to allow intracellular persistence.

### 5.1. Effector Proteins Modulating RIG-I Pathway

**Figure 2:**
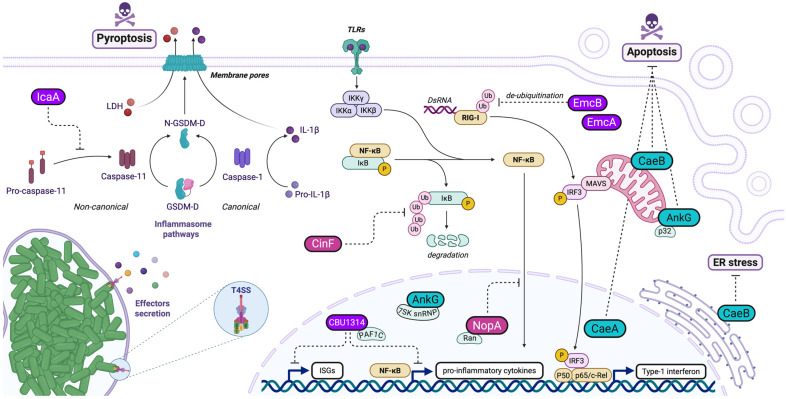
Modulation of the host innate immune systems.

EmcA and EmcB are two effector proteins secreted by the bacterium *Coxiella burnetii* via its Dot/Icm secretion system (Figure 2). EmcB is encoded by a gene conserved in the *Coxiella* spp. environmental metagenome and has specific motifs that confer ubiquitin protease activity. Its structure is adapted to efficiently recognize and cleave ubiquitin bonds. EmcB is described as a cysteine-specific protease that targets long K63-linked ubiquitin chains, a key post-translational modification for the immune receptor RIG-I activation. RIG-I is activated by double-stranded RNAs in the cytosol of infected cells and stimulates the production of type I interferons (IFN-I). EmcB’s activity interferes with the assembly of RIG-I filaments, an essential modification for the activation of this receptor. The activity and function of EmcA are still ill-defined, however, it is involved in blocking RIG-I signaling downstream of EmcB, indicating an evolutionary adaptation to interfere with host defenses [31]. EmcA and EmcB appear to act synergistically, suggesting cooperation in inhibiting IFN-I-dependent immune signaling. These proteins enable *Coxiella* to evade the innate immune response by blocking the activation of RIG-I and, consequently, the production of cytokines such as IFN-I. Suppression of this response prevents host cells from limiting intracellular replication of the bacteria, thereby promoting infection [31]. *Coxiella burnetii* is not the only bacterium that modulates RIG-I signaling. *Legionella pneumophila*, a related bacterium, activates RIG-I by a similar mechanism but does not possess the same inhibitory proteins as *Coxiella*. However, viral proteins such as NS1 from influenza A or ORF64 from Kaposi’s herpes virus share a deubiquitinase function with EmcB [31,87,88]. In summary, the EmcA and EmcB proteins are crucial for the ability of *Coxiella burnetii* to modulate the host immune response and establish persistent infection by inhibiting the RIG-I signaling pathway [31].

Schematic illustration of *Coxiella* manipulation of the host innate immune responses and cellular death pathways during infection. Effector proteins such as CaeA, CaeB, and AnkG actively inhibit both intrinsic and extrinsic apoptotic pathways (blue), with CaeB being particularly effective at blocking mitochondrial-mediated apoptosis. The NF-κB pathway is inhibited through multiple effectors and mechanisms (pink): NopA interacts with Ran-GTPase, disrupting nucleocytoplasmic transport and preventing the nuclear translocation of NF-κB transcription factors, while CinF interferes with the proteasome-mediated degradation of IκBα, maintaining NF-κB in an inactive cytoplasmic state. In addition to NopA, the nuclear-localizing effector CBU1314 targets the PAF1 complex, resulting in the inhibition of NF-κB-, MAPK-, and type I IFN-dependent gene expression. Other immune pathways are impacted by *Coxiella* effectors (purple): The RIG-I pathway is suppressed by EmcA and EmcB. The latter functions as a deubiquitinase that removes activating ubiquitin chains from RIG-I, consequently reducing type I interferon production. Furthermore, the non-canonical inflammasome pathway is subverted by IcaA, which inhibits caspase-11 activation and subsequent pyroptosis. Through the coordinated action of these T4SS-secreted effectors, *Coxiella* effectively dampens multiple host defense mechanisms, establishing itself as a stealth pathogen.

### 5.2. Effector Proteins Interfering with NF-KB Pathway

The nuclear factor kappa-light-chain-enhancer of activated B cells (NF-κB) pathway is a crucial signaling mechanism involved in regulating immune responses, inflammation, cell survival, and proliferation. This pathway is heavily targeted by bacterial pathogens during their infectious cycle [89,90,91]. *Coxiella* secretes several effector proteins capable of altering this pathway (Figure 2).

CinF (CBU0513) is a protein similar to fructose-1,6-bisphosphate (FBP) aldolase/phosphatase, an enzyme involved in sugar metabolism, but has evolved to act as a protein-specific phosphatase to specifically target IκBα [17]. By dephosphorylating IκBα, CinF prevents its degradation and inhibits the nuclear translocation of the NF-κB subunit p65, thus inhibiting the NF-κB signaling pathway [17]. This prevents the production of important cytokines such as IL-1β, IL-6, IL-12, and TNF-α, thus limiting the body’s ability to fight infection. By reducing the host immune response, CinF enables *Coxiella burnetii* to survive and replicate in an intracellular environment while evading host defense mechanisms. This plays a key role in the immune evasion of the bacterium and promotes its persistence in infected cells [17]. In addition, pathogens such as *Legionella pneumophila* and *Shigella flexneri* [92,93,94] use similar mechanisms to inhibit the NF-κB pathway, but they do not have exactly the same proteins or modes of action as CinF [17]. In conclusion, CinF is central to *Coxiella burnetii*’s strategy of evading the host immune response. By inhibiting the NF-κB pathway, it effectively blocks inflammation and allows the bacterium to thrive in infected cells. Interestingly, CinF has been shown to participate in the recruitment of the autophagy protein LC3 to CCVs, which suggests that it might modulate host cellular functions at different levels, ensuring the bacterium’s survival in a hostile environment [17,18].

Another essential *Coxiella burnetii* factor for immune evasion is NopA (CBU1217), which perturbs the transport of the transcription factors p65 and IRF3 to the nucleus [27]. NopA possesses four Regulator of Chromosome Condensation (RCC) repeats, homologous to those found in the eukaryotic Ran protein guanylic nucleotide exchange factor (GEF) RCC1. This structural feature enables NopA to interact directly with Ran [27]. NopA sequesters Ran-GTP in the nucleolus of infected cells, affecting nucleocytoplasmic transport. In turn, this perturbs the nuclear relocalization of transcription factors to the nucleus in response to infections, thereby reducing the production of pro-inflammatory cytokines (TNF-α and IL-8) and attenuating the host immune response [27]. Of note, cytoplasmic retention of p65 is also used by other pathogens, including *Salmonella* (with the SpvD protein) and *Orientia tsutsugamushi* (with the Ank1 and Ank6 effectors) [95,96].

The *Coxiella* effector protein CBU1314 contains nuclear localization signals (NLS) that enable it to enter the nucleus. There, it interacts with the PAF1 complex (PAF1C), a key transcriptional regulator in immune cells. PAF1C is responsible for regulating transcriptional elongation by RNA polymerase II and the expression of inflammatory genes. By associating with PAF1C, CBU1314 blocks the transcription of numerous genes essential to the immune response, including those stimulated by type I interferons, used in antiviral defenses [28,29]. CBU1314 inhibits both the NF-κB and MAPK signaling pathways, which are crucial for the activation of inflammatory responses via innate immunity receptors such as TNF, TLR2, and IL-1β. This leads to a reduction in the expression of inflammation-related genes (such as TNF, IL6, and IL12B) in immune cells. By inhibiting these pathways, CBU1314 suppresses the production of pro-inflammatory cytokines, allowing *Coxiella* to replicate without being destroyed by the immune system [28,29]. Hence, by disrupting PAF1C, CBU1314 not only inhibits the expression of NF-κB-stimulated genes but also prevents interferon-mediated antiviral responses. This allows *Coxiella* to replicate more easily inside host cells, evading an effective immune response [28,29]. Some viral proteins, such as NS1 in influenza and NS5 in dengue, use a similar mechanism by also antagonizing the PAF1C complex [97,98] to block the expression of genes stimulated by interferons, highlighting a strategy shared by *Coxiella* and viruses to evade host defenses [29].

In addition, studies have shown that *Coxiella* survives in the intracellular environment by neutralizing oxidative stress via the enzyme SdrA and modulating IL-17 signaling pathways to evade immune defenses [99]. IL-17 produced by activated CD4^+^ lymphocytes plays a key role in inflammation by activating the NF-κB and MAPK pathways, leading to the production of cytokines such as IL-6 and IL-8 and the recruitment of neutrophils essential for the elimination of *Coxiella* [100,101].

## 6. Manipulation of Host Cell Death

Cell death is a crucial biological process that maintains tissue balance, removes damaged or infected cells, and shapes organism development. It occurs through various pathways, primarily categorized into programmed (regulated) and non-programmed (unregulated) forms. Key types of programmed cell death include apoptosis, necroptosis, pyroptosis, autophagy-dependent cell death, and ferroptosis, each with distinct molecular and morphological features. *Coxiella* secretes effector proteins that interfere with cell death regulation, prolong its survival, and promote infection spread. *Coxiella* manipulates both intrinsic and extrinsic apoptosis pathways and targets pyroptosis, a pro-inflammatory death mechanism (Figure 2). This immune evasion strategy enables the pathogen to establish and maintain a replicative niche inside a modified phagolysosomal compartment, supporting long-term survival and intracellular replication while avoiding detection and clearance by the host immune system. Understanding how bacterial pathogens like *Coxiella* manipulate host cell death pathways offers insights into infection biology and may help identify new therapeutic strategies to combat bacterial diseases.

### 6.1. Anti-Apoptotic Effectors in Coxiella burnetii Pathogenesis

The *Coxiella* effector AnkG (CBU0781) inhibits apoptosis by binding to mitochondrial p32 and uses Importin-α1 for nuclear translocation [102]. This is critical for *Coxiella*’s persistence and replication within host cells. Mutations in *ankG* disrupt its function and trafficking [22,102]. Genetic variability in AnkG leads to functional differences across *Coxiella* strains. The *Nine Mile* reference strain produces a full-length AnkG protein with strong anti-apoptotic effects. In contrast, variants with deletions or mutations exhibit reduced or absent anti-apoptotic activity. Despite these differences, all variants localize to the nucleus and interact with p32 and Importin-α1, though their effects on apoptosis differ [103]. AnkG also interacts with DDX21 and the 7SK snRNP complex, influencing host cell transcription and further modulating apoptosis-related genes [23]. These interactions highlight AnkG’s role in regulating host processes to support *Coxiella* survival.

In addition to AnkG, *Coxiella* employs multiple effector proteins to suppress apoptosis, demonstrating functional redundancy in its immune evasion strategy. Both CaeA (CBU1524) and CaeB (CBU1532) play key roles in inhibiting apoptosis at the mitochondrial level, independent of the p32 protein [32]. CaeA inhibits both intrinsic and extrinsic apoptosis, likely at the intersection of these pathways, through a yet unknown molecular mechanism. When expressed in mammalian cells, CaeA localizes to the host cell nucleus. Its structure includes a predicted coiled-coil region and two nuclear localization signals (NLSs). Sequence analysis of *Coxiella* strains shows genetic variation in *caeA*, particularly in the region encoding a crucial glutamic acid/lysine (EK) tandem repeat motif. The number of EK repeats—three, four, or six—appears critical for CaeA’s strong anti-apoptotic activity [33]. CaeA prevents the cleavage of executioner caspase-7 but does not affect initiator caspase-9. CaeA also upregulates survivin, an inhibitor of apoptosis protein (IAP), though its anti-apoptotic effect is independent of this upregulation. Conversely, CaeB blocks apoptosis more efficiently than CaeA [32,104]. *Coxiella* lacking CaeB shows reduced virulence in *Galleria mellonella* models, with decreased replication, smaller CCVs, and increased host survival [104]. Studies indicate that CaeB acts downstream of Bax and upstream of caspase-9, preventing mitochondrial outer-membrane permeabilization (MOMP)—a critical event in intrinsic apoptosis [104]. CaeB does not interfere with Bax trafficking to the mitochondria or with levels of BCL-2 family proteins, including anti-apoptotic BCL-2 and pro-apoptotic BH3-only proteins. While CaeB was initially reported to localize to mitochondria and the endoplasmic reticulum (ER) during infection, the precise mechanism by which it blocks MOMP remains unclear. The existence of at least three different *Coxiella* anti-apoptotic effector proteins indicates functional redundancy for the inhibition of apoptosis, suggesting that this is a vital feature for the intracellular replication of *Coxiella*.

### 6.2. Anti-Pyroptotic Mechanisms in Coxiella burnetii Pathogenesis

*Coxiella burnetii* utilizes the effector protein IcaA, secreted via its Dot/Icm type IV secretion system, as a key factor in subverting host innate immune responses, specifically by targeting inflammasome activation pathways [36]. IcaA specifically disrupts the non-canonical pathway by preventing caspase-11 activation, which is essential for the detection of cytosolic lipopolysaccharide (LPS) and subsequent gasdermin D-mediated pore formation. This blockade halts the release of pro-inflammatory cytokines (IL-1β and IL-18) and the induction of pyroptotic cell death. IcaA indirectly inhibits the NLRP3 inflammasome-dependent activation of caspase-1, the protease responsible for the maturation and secretion of the pro-inflammatory cytokines. Experimental evidence shows that expression of *icaA* in surrogate bacteria (such as *Legionella pneumophila*) is sufficient to inhibit caspase-11 activation in macrophages, while *C. burnetii* mutants lacking *icaA* fail to suppress caspase-11-dependent inflammasome activation and cytokine release. Thus, IcaA enables *C. burnetii* to evade immune detection by simultaneously targeting multiple inflammasome pathways at the level of caspase activation, thereby preventing both cytokine maturation and pyroptosis and promoting bacterial intracellular persistence.

## 7. Concluding Remarks

Over the past two decades, the study of *Coxiella burnetii* has evolved from a genomic curiosity to a rich field illuminating some of the most sophisticated mechanisms of bacterial subversion known to date. As this review highlights, *Coxiella* uses an extensive repertoire of type IV secretion system (T4SS) effectors to hijack virtually every aspect of host cell biology—from vesicular trafficking, lipid metabolism, and autophagy to immune signaling, cell death pathways, and nuclear transcription. These effectors collectively enable the bacterium to establish and maintain the *Coxiella*-containing vacuole (CCV), a unique lysosome-derived niche where it can replicate undisturbed by host defenses.

In the context of antimicrobial resistance emergence, targeting the T4SS and its cognate translocated effectors is emerging as a promising therapeutic strategy. Indeed, this approach would disarm pathogens rather than kill them directly. By interfering with secretion systems, it is possible to block infection processes or hinder the spread of resistance without exerting strong selective pressure for survival. T4SS inhibitors may limit both host cell manipulation and horizontal gene transfer, thus attenuating virulence. Several natural products and synthetic molecules have been identified that impair secretion system assembly or activity, and some serve as chemical probes to unravel the underlying biology [105]. The strategy of targeting secretion systems represents a shift towards antivirulence therapies. By neutralizing bacterial communication and pathogenic functions rather than viability, these approaches may complement existing antibiotics and help slow the advance of resistance.

Unlike other bacterial pathogens, *Coxiella* has perfected a “stealth” infection strategy, suppressing or evading host innate immune recognition and actively preventing host cell death so that the infected cell remains a stable incubator for its prolonged replication. This contrasts sharply with paradigms like *Legionella pneumophila*, which uses a similar Dot/Icm type IV secretion system but aggressively rewires the host to create a short-term, ER-like niche at the expense of normal endolysosomal trafficking. *L. pneumophila* effectors largely serve to disable autophagy and endosomal maturation, allowing the bacterium to avoid destruction in lysosomes [106], whereas *Coxiella* effectors embrace and even co-opt the endolysosomal pathway, for example by recruiting autophagic membranes, to expand a spacious, lysosome-derived vacuole. Similarly, *Chlamydia trachomatis* occupies a membrane-bound inclusion and secretes its own arsenal of effectors to insulate that compartment from immune detection and lysosomal fusion [106]. In an even more divergent approach, *Salmonella enterica* injects dozens of type III–secreted effectors that not only mediate intracellular survival but also actively trigger pro-inflammatory signaling in the host, leveraging inflammation as part of its life cycle [106]. Together, these comparisons underscore the remarkable evolution of effector–host interactions across intracellular bacteria: Some pathogens incite and then subvert host defenses, whereas *Coxiella* exemplifies a quieter trajectory, one that prioritizes immune evasion and host cell retention over rapid host damage.

Studying how *Coxiella* co-opts host functions for its survival can illuminate general principles of coadaptation. Many *Coxiella* effectors carry eukaryotic-like domains, indicating they were acquired via horizontal gene transfer from hosts. This not only underscores how effectors evolve by pirating host genes but also provides a window into the evolution of host/pathogen interactions, helping us reconstruct how pathogens innovated by repurposing eukaryotic activities. This rare evolutionary leap from mutualist to pathogen offers a powerful framework to study the tipping points of pathogenesis. By retracing the genetic changes (e.g., secretion system acquisition, effector expansion) that turned a harmless symbiont into a pathogen, we can better understand the evolutionary paths leading to virulence. *C. burnetii* thereby stands as a case study in the continuum between symbiosis and disease, demonstrating how shifts in host or environment can drive pathogens to either attenuate or amplify their harm.

## Figures and Tables

**Table 1:** *Coxiella* effectors with identified cellular partners and/or functions.

Gene (CDS)	Acronym	Cellular Function and Target (If Known)	Reference
CBU0021	CvpB/Cig2	Interacts with phosphatidylinositol 3-phosphate (PI3P), inhibits PIKfyve activity and stimulates PI3P and LC3 recruitment to the CCV, participates in Cathepsin B removal from the CCV	[3,4,5,6,7]
CBU0041	CirA/CoxCC1	Stimulates RhoA GTPase activity	[8,9]
CBU0077	MceA	Is farnesylated by the host cell and localizes to the mitochondrial outer membrane	[10]
CBU0122	CvpM	Localizes to Mitochondria and CCV	[11]
CBU0175	CstK	Interacts with TBC1D5 and displays threonine and tyrosine kinase activity	[12]
CBU0388	CetCb2	Enhances MAP kinase pathway in yeast	[13]
CBU0425	CirB	Interacts with proteasomal subunit PSMB5 and inhibits host proteasome activity	[9,14]
CBU0447	AnkF	Interacts with and recruits vimentin to the CCV	[15,16]
CBU0513	CinF	Displays phosphatase activity and dephosphorylates IkBα	[17,18]
CBU0626	CvpF	Recruits RAB26 to the CCV	[19]
CBU0635		Interferes with host protein secretion	[16,20]
CBU0665	CvpA	Interacts with clathrin adaptor complex AP2	[16,21]
CBU0781	AnkG	Interacts with p32 (gClqR), DDX21 and 7SK snRNP to inhibit apoptosis and participate in transcriptional reprogramming	[22,23,24]
CBU0937	MceB/CirC	Interacts with mitochondrial protein CYP1B1	[9,25,26]
CBU1217	NopA	Interacts with Ran to perturb nucleocytoplasmic trafficking and innate immune signaling	[27]
CBU1314	coxCC6	Interacts with PAF1C to inhibit NF-κB-, MAPK-, and type I IFN-dependent gene expression	[28,29]
CBU1370	*Cb*EPF1	Interacts with lipid droplets	[30]
CBU1387	EmcA/Cem6	Inhibits RIG-I signaling	[31]
CBU1425	MceC	Interacts with mitochondrial protein YME1L	[25]
CBU1524	CaeA	Inhibit intrinsic apoptosis pathway	[32,33]
CBU1532	CaeB	Inhibit intrinsic apoptosis pathway	[32]
CBU1751	Cig57	Interacts with FCHO2 to co-opt clathrin-mediated trafficking and autophagy	[3,34,35]
CBU1823	IcaA	Inhibits non-canonical inflammasome	[36]
CBU1863	CvpE	Interacts with Phosphatidylinositol 3-phosphate (PI3P), perturbs PIKfyve activity and suppresses lysosomal calcium transient receptor potential channel mucolipin 1 (TRPML1) activity	[4,37]
CBU2007	Vice	Interacts with LBPA/CHMP3/ALIX, stimulates macropinocytosis and inhibits ESCRT machinery	[38]
CBU2013	EmcB	Displays ubiquitin-specific cysteine protease activity and inhibits RIG-I signaling	[31]
CBUA0013	CpeB	Promotes LC3-II accumulation and contributes to virulence in SCID mouse model	[14,16,39]

**Table 2:** *Coxiella* T4-secreted proteins with no identified cellular functions.

Gene (CDS)	Acronym	Reference
CBU0072	AnkA	[16]
CBU0080		[16]
CBU0129		[16]
CBU0145		[16]
CBU0201	AnkC	[27]
CBU0295		[16]
CBU0329		[16]
CBU0410	Cig12	[16]
CBU0414	CoxH1	[16,18]
CBU0505	Cig14	[27]
CBU0519	DedA	[27]
CBU0534		[4,16]
CBU0542	LigA	[27]
CBU0547		[27]
CBU0794		[16]
CBU0885	CetCb4	[4,16]
CBU0978	Cem3	[18]
CBU1024		[16]
CBU1045		[16]
CBU1107		[16]
CBU1108		[16]
CBU1198		[11]
CBU1213	AnkI	[27]
CBU1366	Cig40	[27]
CBU1457	Cig43	[27]
CBU1460		[16]
CBU1461		[16]
CBU1493		[4,16]
CBU1525		[16]
CBU1530		[40]
CBU1543		[4]
CBU1556	CvpC	[4,16]
CBU1569		[16]
CBU1594	MceD	[25]
CBU1614		[16,40]
CBU1676		[4,16]
CBU1677	MceE	[25]
CBU1685		[40]
CBU1686		[40]
CBU1724	CetCb6	[27]
CBU1752		[40]
CBU1776		[16]
CBU1780		[3]
CBU1790		[16]
CBU1799		[27]
CBU1818	CvpD	[4,16]
CBU1819		[4,16]
CBU1825		[16]
CBU1863		[16]
CBU1963		[16]
CBU2028		[18]
CBU2052	CirD	[9,16]
CBU2056		[16]
CBU2059	CirE	[9]
CBUA0006	CpeA	[39]
CBUA0014	CpeC	[16,39]
CBUA0015	CpeD	[16,39]
CBUA0016	CpeE	[16,39]
CBUA0023	CpeF	[39]

## Data Availability

Not applicable.
